# Application of COI Sequences in Studies of Phylogenetic Relationships Among 40 Apionidae Species

**DOI:** 10.1673/031.012.1601

**Published:** 2012-02-03

**Authors:** Aneta A. Ptaszyńska, Jacek Łętowski, Sebastian Gnat, Wanda Małek

**Affiliations:** ^1^ Department of Botany and Mycology, M. Curie-Skłodowska University, 19 Akademicka St., 20-033 Lublin, Poland; ^2^ Zoology Department, University of Life Sciences in Lublin, 13 Akademicka St.; 20-033 Lublin, Poland; ^3^ Department of Genetics and Microbiology, M. Curie-Skłodowska University, 19 Akademicka St., 20-033 Lublin, Poland

**Keywords:** systematics, phylogenetics, lnsecta, Curculionoidea, weevils

## Abstract

The systematics of the family Apionidae, as well as the superfamily Curculionoidea, is currently in a state of flux. The comparative analyses of COI sequences from our studies shed some light on the systematics of these weevils. To study the relationship among the organisms of the family Apionidae, we determined the COI sequences of representatives of 23 species and 15 genera, i.e., *Apion, Betulapion, Catapion, Ceratapion, Cyanapion, Eutrichapion, Exapion, Hemitrichapion, Holotrichapion, Ischnopterapion, Protapion, Pseudoperapion, Psudoprotapion, Pseudostenapion*, and *Stenopterapion.* Then, they were compared with the COI sequences of 19 species and eight genera from GenBank (*Aspidapion, Ceratapion, Exapion, Ischnopterapion, Lepidapion, Omphalapion, Oxystoma*, and *Protapion*). The phylogenetic relationships inferred from molecular data are similar to the classification system developed by Alonso-Zarazaga and Lyal (1999), with some exceptions within the tribe Oxystomatini, and genera *Ceratapion* and *Exapion.*

## Introduction

Beetles are among the most diverse group of animals on the planet, which contains more species than any other order in the animal kingdom. They comprise 25% of all known life—forms (Beckmann 2004). The most numerous beetle group is the weevils, classified to the superfamily Curculionoidea. It contains about 62,000 described species and 6000 genera (Thompson 1992; Kuschel 1995; Gønget 2004; Oberprieler et al. 2007). Most weevils are plant—feeding both as larvae and adults, and utilize every plant part of almost all plant taxa. Furthermore, many of these beetles are crop pests (Anderson 1995). Additionally, the taxonomic groups of weevils are often restricted to particular host groups, e.g., to conifers, cycads, dicots, or monocots, or even to their subsets, although there are many exceptionally polyphagous species.

Among weevils, the family Apionidae is represented by 1900 species of small, pear—shaped beetles, hence the name of the type genus (Greek *apion*: a small pear). They are distinguished from other weevils by long trochanters and straight, non—geniculate antennae (except for very few species with a different structure). The majority of the species live on Fabaceae and Asteraceae, very few on Polygonaceae, and single species feed on representatives of other plants (Salicaceae, Betulaceae).

The systematics of the family Apionidae has been previously studied by Wagner (1926), Smreczyński (1965), Dieckmann (1977), and Alonso-Zarazaga (1990). When compiling the taxonomy for Winkler's catalogue (1927– 1932), Wagner (1926) introduced 57 subgenera. Smreczyński's key (1965) for identification of weevils adopted the division developed mainly by Schilsky (1901, 1906) and Reitter (1916), with amendments made by Saint-Claire Deville (1924) and Hoffmann (1958). The author of this study distinguished one genus, *Apion* Herbst (1797), containing 26 subgenera. Similarly, in the monograph of Apioninae, Dieckmann (1977) distinguished one genus *Apion* containing 38 subgenera. Smreczyński (1965) regarded this division to be too far—reaching, since it separated natural groups. Alonso-Zarazaga (1990) raised the subgenera to the genus level, and divided Apionidae into 37 genera.

All taxonomic classifications of Apionidae are mainly based on morphological features, and the recent ones also take into account the distribution and biology of individual species (Wagner 1926; Smreczyński 1965; Dieckmann 1977; Alonso-Zarazaga 1990). Nevertheless, the systematics of most of the weevil taxa is still controversial (Hundsdoerfer et al. 2009). First, the systematic level of Apionidae/Apioninae varies from the family to subfamily level. Cladistic analysis of Curculionoidea proposed by Kuschel (1995) located Apioninae inside the family Brentidae, while Alonso-Zarazaga and Lyal (1999) classified Apionidae as a family. However, in their summarizing work, Oberprieler et al. (2007) distinguished Apioninae as a subfamily of the family Brentidae (Brentidae: Ithycerinae, Microcerinae, Eurhynchinae, Brentinae, Apioninae, and Nanophyinae). Secondly, the relationships among Apionidae tribes are controversial. In Hundsdoerfer et al. (2009), only Aplemonini, Kalcapiini, Malvapiini, and Piezotachelini tribes formed a monophylum. Lastly, some species locations in the genera changed from Wagner (1926) to Alonso-Zarazaga (1990), e.g., Eutrichapion classified by Wagner (1926) as a subgenus included eight species, whereas in Smreczyński's key (1965) there were 21 species; in Dieckmann (1977) there were three species, while AlonsoZarazaga (1990) raised Eutrichapion to the genus level and included 10 species.

Molecular markers provide a useful means to obtain additional information on phylogenetic relationships among closely related species. Caterino et al. (2000) selected four DNA markers to promote synergy among the phylogenetic research of COI, large mitochondrial ribosomal subunit (16S rRNA), elongation factor 1 alpha (*EF-1α*), and small nuclear ribosomal subunit (18S rRNA). Such investigations based on a combined analysis of 18S rDNA and morphological data were conducted by Marvaldi and Morrone (2000) and Marvaldi et al. (2002), and resulted in identification of seven weevil families (Anthribidae, Attelabidae, Belidae, Brentidae including Apioninae, Caridae, Curculionidae, and Nemonychidae). A study of the phylogeny of the Curculionoidea was published by Hundsdoerfer et al. (2009). The mitochondrial gene COI was successfully used in similar investigations of other beetles (Langor and Sperling 1997; Caterino et al. 2000; Sequeira et al. 2000; Moya et al. 2006). Moreover, the latest studies have indicated that the COI gene is as efficacious for resolving the phylogenetic relationships among closely related species as more rapidly evolving genes like *ND2* (Lu et al. 2011). Nowadays, the COI gene sequence is one of the most widely used genetic marker for resolving the phylogenetic relationships of insects.

The present study provided preliminary information to estimate the relationship among 40 species from 19 genera (seven tribes) of Palaearctic Apionidae and verified the conformity of a COI phylogeny with the Apionidae classification systems based mainly on morphological and biological features, especially those developed by AlonsoZarazaga and Lyal (1999).

## Materials and Methods

### Specimen sampling, DNA extraction, PCR sequencing

All the investigated Apionidae species were collected from Poland and were classified according to the classification system of Alonso-Zarazaga and Lyal (1999). Depending on its availability, two to six specimens from each species were chosen for further analyses.

Apionidea are small insects with the length from 1.1–4.3 mm. After freezing at -70 °C, the total genomic DNA was extracted from the head and thorax of each specimen. Before DNA extraction, the weevils were surface sterilized by immersion in 70% ethanol. The risk of DNA contamination with gut content was reduced by removal of abdomens. Beetle remains were deposited in the Zoology Department (University of Life Sciences in Lublin). The total genomic DNA was extracted following the QIAamp® DNA Micro Kit procedure (QIAGEN Inc., www.qiagen.com).

Polymerase chain reaction (PCR) cocktails were prepared using the QIAGEN Taq PCR Core Kit (QIAGEN Inc.) and were carried out in a thermal cycler in 50 µL of cocktails containing 5 µL PCR buffer, 10 µL Q solution, 0.2 mM dNTP mix, 1.5 U Taq DNA polymerase, 0.5 µL of each primer, approximately 0.3 µg of DNA template, and ddH_2_O added to a final reaction volume of 50 µL. For DNA amplification, the following PCR cycling conditions were used: 1 min at 94 °in at 41.5 °d 1 min at 72 ° repeated for 30 cycles, and 10 min at 72 ° PCR products were sequenced using ABI 3100 Avant. Although the annealing temperature was rather low, no unspecific reaction products were observed and the sequencing gave clear and readable results.

A region of 811 bp of the mtDNA COI gene was amplified using primers designed in a Primer3 program (Rozen and Skaletsky 2000): 5′TTTATTCTACCAGGATTTGG3′ and 5′ATTTGGGGTTTAAATCCAATGC3′. These primers amplified the COI gene in all the investigated species of Apionidae and Curculionidae (Curculionidae: Mecinini: *Miarus ajugae*, Herbst 1795; FJ657425-7). The amplified region refers to 2221-303 lnt of the *Drosophila yakuba* COI gene. Amplified sequences of 24 species were released into GenBank (Table 1). To widen our investigation, COI gene sequences of 21 species were obtained from NCBI (Table 2).

### Phylogenetic analysis

The sequences were corrected manually with the aid of Chromas 1.45 (McCarthy 1998). To construct the sequence identity matrix, consensus sequences of each species were aligned using BioEdit (Hall 1999). The alignment regions refer to 2283-2800nt of the *D. yakuba* COI gene.

The COI gene sequences of the studied Apionidae species obtained and those of the related organisms (Apionidae and Nanophyidae) available in the GenBank database were aligned using ClustalX (Thompson et al. 1997) and then were visually corrected with GeneDoc (Nicholas et al. 1997). Phylogenetic trees were constructed using the neighbor joining (NJ) and maximum likelihood (ML) methods. In the NJ method, the phylogenetic distances were estimated with Kimura's two—parameter model (K2P) (Kimura 1980) using the MEGA 4 program (Tamura et al. 2007). ML analysis was performed with PhyML version 3.0, after determining the appropriate nucleotide substitution model selected by Akaike information criterion (AIC) in PAUP version 4.0b10 software, using MODELTEST 3.7 (Posada and Crondall 1998). The optimal evolution model used for the analyzed sequences was TVM+I+G (Transversion Model with Invariant sites and a Gamma rate distribution). Weevil clusters, strongly supported by the NJ distance method, were in agreement with the ML analysis and vice versa; therefore, only ML—based trees are presented in this paper.

Robustness of tree branches was determined by bootstrap analysis using 100 re—samplings. The phylogram was presented in the Tree View program (Page 1996). A complementary sequence of *Lepidiota albistigma* (Coleoptera: Scarabaeidae), (DQ524367, Ahrens et al. 2007) was used as the outgroup.

## Results

Genetic diversity within or among populations in comparison to that which occurs among species is usually very low; consequently, in all the investigated COI sequences obtained from Apionidae belonging to one species, the differences among sequences were not large. Moreover, the level of divergence was independent of the specimen locality. The studied sequences of beetles deriving from Turkey, Italy, Greece, France (Antonini et al. 2009), Britain (Hunt et al. unpublished), and Poland belonging to one species were almost identical. The 518nt long COI gene fragment indicated sequence similarity from 95.1% to 100%. The *COI* sequence similarity among the species of one genus was in the range from 83% to 92% (Table 3). Such a sequence similarity rate allows even closely related species to be distinguished.

**Figure 1.**
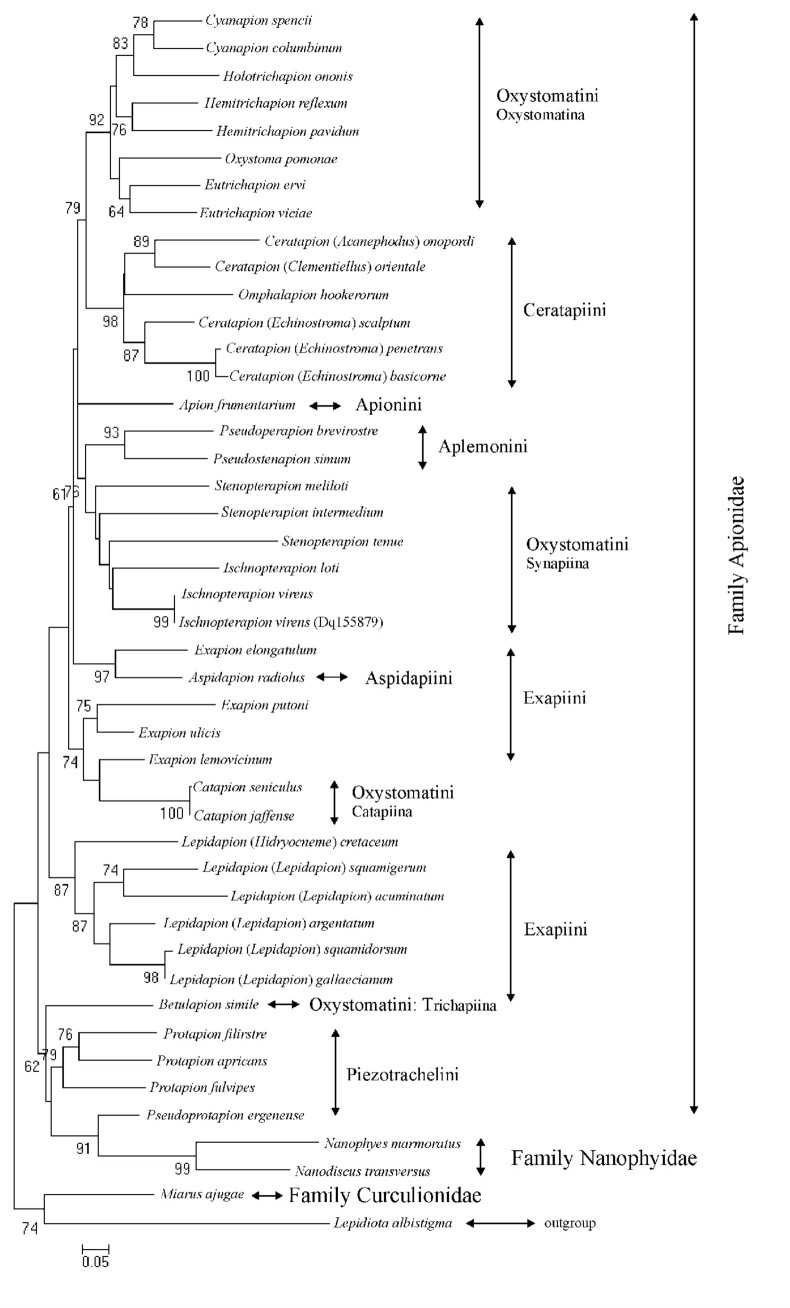
The phylogenetic tree based on analysis of COI gene sequences, constructed using the maximum likelihood method (ML) in PhyML version 3.0, after determining the appropriate nucleotide substitution model selected by Akaike information criterion (AIC) in PAUP version 4.0b10 software, using MODELTEST 3.7. Bootstrap values > 60% are indicated on the nodes. The scale line represents evolutionary changes. High quality figures are available online.

The phylogenetic tree based on the mitochondrial COI gene sequences of 42 species (Figure 1) shows an arrangement of investigated species, which is in agreement with the classification system of AlonsoZarazaga and Lyal (1999). Generally, species belonging to the same genus are grouped together with few exceptions; species belonging to the genus *Exapion* (tribe Exapiini) form two clusters, first with *Aspidapion radiolus* (tribe Aspidapiini) and the other one with *Catapion jaffense* and *C. seniculus* (tribe Oxystomatini). Both these clusters are separated from *Lepidapion* species, which also belong to the tribe Exapiini.

Similarly, species of the genus *Ceratapion* (subgenus *Echinostroma*) are grouped together and separated from *C. (Acanephodus)* *onopordi* and *C. (Clementiellus) orientale* by *Omphalapion hookerorurm.*

In our studies, *Lepidapion (Hidryocneme) cretaceum* is outside the group comprising *Lepidapion (Lepidapion)* species, following the classification system of Alonso-Zarazaga and Lyal (1999).

Four subtribes of the tribe Oxystomatini (Oxystomatina, Synapiina, Catapiina and Trichapiina) do not form one group, but instead are separated from each other. In the light of the molecular data, the tribe Oxystomatini seems to be non—monophyletic and the subtribes included into it are less related to each other than to the other tribes of the family Apionidae. Undoubtedly, analysis of the relationships of species belonging to the tribe Oxystomatini needs more attention.

Similarly, the correlations between the families Nanophyidae and Apionidae need more consideration. Two of the studied Nanophyidae species are grouped with species from the tribe Piezotrachelini (family Apionidae) with a high bootstrap value.

## Discussion

Phytophagan beetles are a relatively diverse group of organisms that probably originated during the early Jurassic. Differentiation of these insects is correlated with the diversification of conifers at this period of time (Carpenter 1992; Zherikhin and Gratshev 1995; Gratshev and Zherikin 2003, Hunt et al. 2007). Among beetles, weevils gained a great evolutionary success connected with their specialized endophytophagy (larvae developing inside a great variety of plant structures). Despite the huge number of weevil species, recent studies based on molecular as well as morphological data provided evidence for the monophyly of the superfamily Curculionoidea (Marvaldi 2005; Vogler 2005; Hunt et al. 2007, Wanat 2007). The major groups of weevils at the family and subfamily level are relatively well established, except for the large and phylogenetically complex family Curculionidae. Classification of this taxon is problematic and there is still disagreement as to its phylogeny or even cogency of most groups within it. According to Oberprieler et al. (2007), e.g., the recent catalog of Curculionidae genera (AlonsoZarazaga and Lyal 1999) is an “amalgamation of not evidently closely related genera” and subfamilies grouped together only due to consideration for their traditional units.

Although there are many publications describing the relationships among weevil families, there are few studies that investigate lower taxonomic units. Wanat (1995) published interesting work on the systematics and phylogeny of the tribe Ceratapiini. In the same year, Zherikhin and Gratshev (1995) described patterns in the wing arrangement within the Apionidae, which may be phylogenetically important, e.g., authors suggested that wings of Metapiini, *Pseudaplemonus*, and *Exapion* have similar wing venation. The authors also found that some tribes established by Alonso-Zarazaga (1990) are heterogenous. They noticed that *Pseudopirapion* differs from other Piezotrachelini in wing venation. Genera placed in Oxystomatini are dissimilar in wing characters and *Pseudaplemonus* has nearly no important common features with other Aplemonini. Similar doubts as to homogeneity of the tribe Oxystomatini can be found in our results. The tribe Oxystomatini emerges as a very heterogeneous and diverse one. Even its division into subtribes needs careful consideration.

The phylogenetic relationships of the weevils studied inferred from the mitochondrial DNA sequences compared to Apionidae systematics established by Winkler (1927–1932), Smreczyński (1965), Dieckmann (1977), and Alonso-Zarazaga and Lyal (1999) indicate that the classification system proposed by Alonso-Zarazaga and Lyal (1999) is the most accurate. There are tribes like Aplemonini (supported in our studies by two species) and Piezotrachelini (four species) that form a monophyletic group in both the present study and in Hundsdoerfer et al. (2009); however, in accordance with Hundsdoerfer et al. (2009) and Alonso-Zarazaga and Lyal (1999), some exceptions were also found. The tribe Oxystomatini needs reexamination. The phylogenetic relationships inferred from the analyses of COI sequences indicate that the tribe Oxystomatini is non—monophyletic, and the subtribes it includes such as Oxystomatina, Synapiina, Catapiina, and Trichapiina appear to be more correlated with other Apionidae tribes than with each other. Therefore, the relationships within the tribe Oxystomatini need further careful consideration. In light of our study, the four subtribes of the tribe Oxystomatini (Oxystomatina, Synapiina, Catapiina and Trichapiina) should be raised to the tribe level.

Genera like *Exapion* and *Ceratapion* also need reexamination because they were not found to be monophyletic in our study. Similar doubts as to monophyly of the tribe Exapiini can be found in Hundsdoerfer et al. (2009), based on an analysis of 16S and 18S rDNA. Molecular data derived from analyses of additional loci (16S rDNA, 18S rDNA, *EF-1α*) as well as careful morphological investigations of further species of these taxa should clarify their systematic position.

The two species of Nanophyidae grouped with Piezotrachelini (Apionidae) suggest that Nanophyidae and Apionidae are closely related and should be placed in one taxon, as claimed by Kuschel (1995) and Oberprieler et al. (2007) but refuted in Hundsdoerfer et al. (2009). However, data from the COI gene analysis are more informative at the genus rather than family level (Wilson 2010); therefore, close relationships between Nanophyidae and Apionidae are not strongly supported in our analysis.

In conclusion, the number of the described species belonging to weevils is estimated at approximately 62,000; the possible total number of species may be 220,000, and among them the family Apionidae includes about 1900 species (Oberprieler et al. 2007). The current classification of these insects is under continuous revision, because of the addition of molecular data and new features derived from adult and especially larval morphology or biology. In our study, the relationships inferred from molecular data of 40 Apionidae species are similar to those in the classification system of Alonso-Zarazaga and Lyal (1999).

**Table 1.**
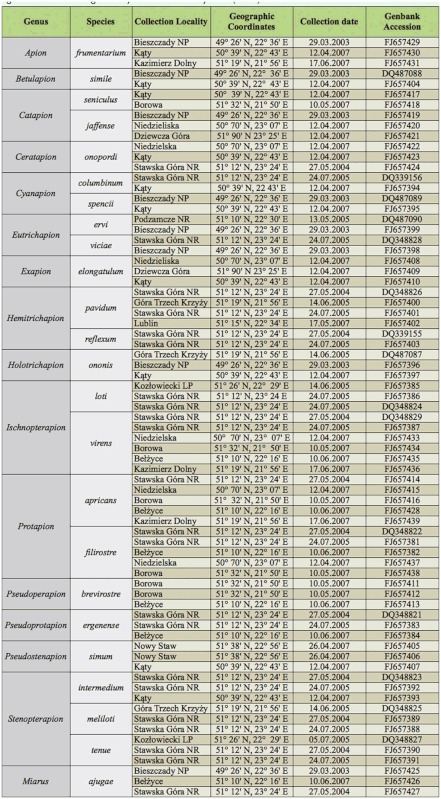
Collection data: LP - Landscape Park; NP - National Park; NR - Nature Reserve. Species classified according to Alonso-Zarazaga and Lyal classification system (1999).

**Table 2.**
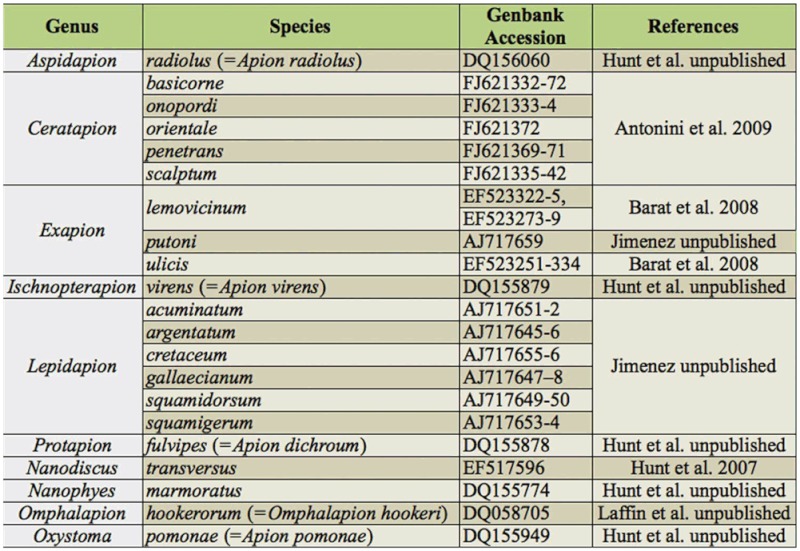
Sequences obtained from NCBI. Species classified according to Alonso-Zarazaga and Lyal classification system (1999); in—parentheses species names from original works.

**Table 3.**
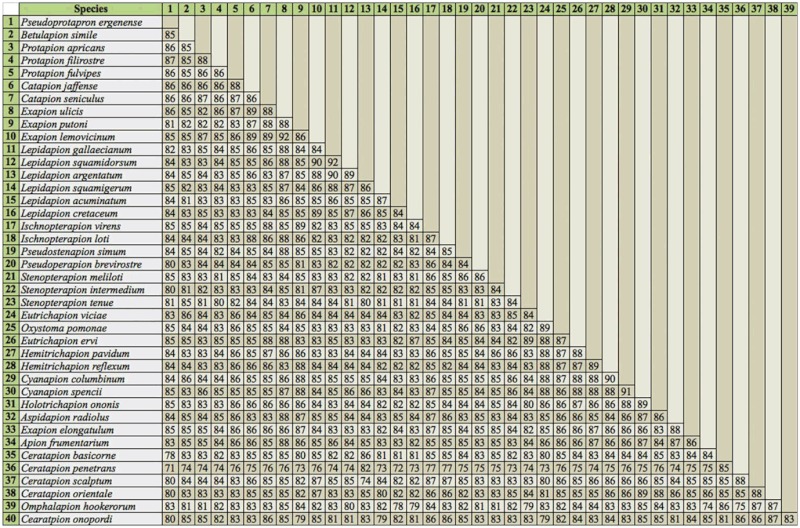
Percentage of sequence identity calculated in BioEdit from 518nt alignments of consensus COI gene sequences of each Apionidae species.

## References

[bibr01] Ahrens D, Monaghan MT, Vogler AP (2007). DNA—based taxonomy for associating adults and larvae in multi—species assemblages of chafers (Coleoptera: Scarabaeidae).. *Molecular Phylogenetics and Evolution*.

[bibr02] Alonso-Zarazaga MA (1990). Revision of the supraspecific taxa in the Palaearctic *Apionidae* Schoenherr, 1823 (Coleoptera, Curculionoidea) 2 Subfamily Apioninae Schoenherr, 1823: Instruction, keys and descriptions.. *Graellsia*.

[bibr03] Alonso-Zarazaga MA, Lyal CHC (1999). *A World Catalogue of Families and Genera of Curculionoidea (Insecta: Coleoptera) (Excepting Scolytidae and Platypodidae).*. Entomopraxis..

[bibr04] Anderson RS (1995). An evolutionary perspective on diversity in Curculionoidea.. *Memoirs of the Entomological Society of Washington*.

[bibr05] Antonini G, Coletti G, Serrani L, Tronci C, Cristofaro M, Smith L (2009). Using molecular genetics to identify immature specimens of the weevil *Ceratapion basicorne* (Coleoptera: Apionidae).. *Biological Control*.

[bibr06] Barat M, Tarayre M, Atlan A (2008). Genetic divergence and ecological specialisation of seed weevils (*Exapion* spp.) on gorses (*Ulex* spp.).. *Ecological Entomology*.

[bibr07] Beckmann P (2004). *Living Jewels: The Natural Design of Beetles Paul Beckmann: Living Jewels: The Natural Design of Beetles*..

[bibr08] Carpenter FM, Moore RC, Kaesler RL (1992). Superclass Hexapoda.. *Treatise on Invertebrate Paleontology*, Part R, Arthropoda 4, v. 3 and 4..

[bibr09] Caterino MS, Cho S, Sperling FAH (2000). The current state of insect molecular systematics: A thriving Tower of Babel.. *Annual Review of Entomology*.

[bibr10] Deville S-CJ (1924). *Faune des Coléoptères du Bassin de la Seine 6 (bis).*. Supplément aux Rhynchophora..

[bibr11] Dieckmann L (1977). Beiträge zur Insektenfauna der DDR: Coleoptera— Curculionidae (Apioninae).. *Beiträge zur Entomologie*.

[bibr12] Gønget H (2004). *The Brentidae (Coleoptera) of Northern Europe on CD—ROM and The Nemonychidae, Anthribidae and Attelabidae (Coleoptera) of Northern Europe*, volumes 34 and 38..

[bibr13] Gratshev VG, Zherikhin VV (2003). The fossil record of weevils and related beetle families (Coleoptera, Curculionoidea).. *Acta Zoologica Cracoviensia*.

[bibr14] Hall TA (1999). BioEdit: a user—friendly biological sequence alignment editor and analysis program for Windows 95/98/NT.. *Nucleic Acids Symposium Series*.

[bibr15] Hoffmann A 1958 *Faune de France*, volume 62. *Coleopteres Curculionides (III Partie).* Federation Française des Societes des Sciences Naturelles.

[bibr16] Hundsdoerfer A, Meier H, Rheinheimer J, Wink M (2009). Towards the phylogeny of the Curculionoidea (Coleoptera): Reconstructions from mitochondrial and nuclear ribosomal DNA sequences.. *Zoologischer Anzeiger*.

[bibr17] Hunt TJ, Papadopoulou A, Vogler AP Barcoding British Beetles. Unpublished (NCBI)..

[bibr18] Hunt T, Bergsten J, Levkanicova Z, Papadopoulou A, John OS, Wild R, Hammond PM, Ahrens D, Balke M, Caterino MS, Gomez-Zurita J, Ribera I, Barraclough TG, Bocakova M, Bocak L, Vogler AP (2007). A comprehensive phylogeny of beetles reveals the evolutionary origins of a superradiation.. *Science*.

[bibr19] Jimenez RY Phylogeny of the genus *Lepidapion.* Unpublished (NCBI)..

[bibr20] Kimura M (1980). A simple method for estimating evolutionary rate of base substitutions through comparative studies of nucleotide sequences.. *Journal of Molecular Evolution*.

[bibr21] Kuschel G (1995). A phylogenetic classification of Curculionoidea to families and subfamilies.. *Memoirs of the Entomological Society of Washington*.

[bibr22] Langor DW, Sperling FAH (1997). Mitochondrial DNA sequence divergence in weevils of the *Pissodes strobi* species complex (Coleoptera: Curculionidae).. *Insect Molecular Biology*.

[bibr23] Lu JM, Li T, Chen HW (2011). Molecular phylogenetic analysis of the *Stegana ornatipes* species group (Diptera: Drosophilidae) in China, with description of a new species.. *Journal of Insect Science*.

[bibr24] Marvaldi AE (2005). Una filogenia preliminar de escarabajos Cucujiformia: existe un clado Phytophaga? (Insecta: Coleoptera).. *Revista de la Sociedad Entomológica Argentina*.

[bibr25] Marvaldi AE, Morrone JJ (2000). Phylogenetic systematics of weevils (Coleoptera: Curculionoidea): a reappraisal based on larval and adult morphology.. *Insect Systematics and Evolution*.

[bibr26] Marvaldi AE, Sequeira AS, O'Brien CW, Farrell BD (2002). Molecular and morphological phylogenetics of weevils (Coleoptera, Curculionoidea): do niche shifts accompany diversification?. *Systematic Biology*.

[bibr27] McCarthy C (1998). *Chromas 1.45.*.

[bibr28] Moya Ó, Contreras-Diaz HG, Oromi P, Juan C (2006). Using Statistical phylogeography to infer population history: Case studies on *Pimelia* darkling beetles from the Canary Islands.. *Journal of Arid Environments*.

[bibr29] Nicholas KB, Nicholas HB, Deerfield DW (1997). GeneDoc: Analysis and Visualization of Genetic Variation.. *EMBNEW News*.

[bibr30] Oberprieler RG, Marvaldi AE, Anderson RS (2007). Weevils, weevils, weevils everywhere.. *Zootaxa*.

[bibr31] Page RD (1996). TREE VIEW: an application to display phylogenetic trees on personal computers.. *Computer Applications in the Biosciences*.

[bibr32] Posada D, Crondall KA (1998). Modeltest: testing the model of DNA substitution.. *Bioinformatics*.

[bibr33] Reitter E (1916). *Fauna Germanica: die Käfer des Deutschen Reiches*.

[bibr34] Rozen S, Skaletsky HJ, Krawetz S, Misener S (2000). Primer3 on the WWW for general users and for biologist programmers.. *Bioinformatics Methods and Protocols: Methods in Molecular Biology*..

[bibr35] Schilsky J (1901). Die Käfer Europas. Nach der Natur beschrieben von Dr. H. C. Küster und Dr. G. Kraatz. Fortgesetzt von J. Schilsky.. Nürnberg, 38, VI + A-K..

[bibr36] Schilsky J (1906). Die Käfer Europas. Nach der Natur beschrieben von Dr. H. C. Küster und Dr. G. Kraatz.. Fortgesetzt von J. Schilsky, Nürnberg, 43, VIII + CXIX+107..

[bibr37] Sequeira AS, Lanteri AA, Scataglini MA, Confalonieri VA, Farrell BD (2000). Are flightless *Galapaganus* weevils older than Galapagos Islands they inhabit?. *Heredity*.

[bibr38] Smreczyński S (1965). *Klucze do oznaczania owadów Polski. Cz. XIX, Zesz. 98a (Coleoptera, Curculionidae).*.

[bibr39] Tamura K, Dudley J, Nei M, Kumar S (2007). MEGA4: Molecular Evolutionary Genetics Analysis (MEGA) software version 4.0.. *Molecular Biology and Evolution*.

[bibr40] Thompson RT (1992). Observations on the morphology and classification of weevils (Coleoptera: Curculionoidea) with a key to the major groups.. *Journal of Natural History*.

[bibr41] Thompson JD, Gibson TJ, Plewnik F, Jeanmougin F, Higgins DG (1997). The CLUSTAL X windows interface: Flexible strategies for multiple sequence alignment aided by quality analysis tools.. *Nucleic Acids Research*.

[bibr42] Vogler A, Beutel RG, Leschen RAB (2005). Molecular systematics of Coleoptera: what has been achieved so far?. *Coleoptera, Beetles. Volume 1: Morphology and Systematics (Archostemata, Adephaga, Myxophaga, Polyphaga partim). Handbook of Zoology*.

[bibr43] Wagner H (1926). *Apion*-Studien II. (Curcl.). Revision des Subgen. Protapion Schilsky. (45. Beitrag zur Kenntnis der Subfam. Apioninae).. *Coleopterologisches Centralblatt*.

[bibr44] Wanat M (1995). Systematics and phylogeny of the tribe Ceratapiini (Coleoptera: Curculionoidea: Apionidae).. *International Journal of Invertebrate Taxonomy*, Suppl. 3..

[bibr45] Wanat M (2007). Alignment and homology of male terminalia in Curculionoidea and other Coleoptera.. *Invertebrate Systematics*.

[bibr46] Wilson JJ (2010). Assessing the Value of DNA Barcodes and Other Priority Gene Regions for Molecular Phylogenetics of Lepidoptera.. *PLoS ONE*.

[bibr47] Winkler A (1927–1932). *Catalogus Coleopterorum Regionis Palaearcticae*.

[bibr48] Zherikhin VV, Gratshev VG, Pakaluk J, Slipinski SA (1995). A comparative study of the hind wing venation of the superfamily Curculionoidea with phylogenetic implications.. *Biology, Phylogeny, and Classification of Coleoptera. Papers celebrating the 80th Birthday of Roy A. Growson.*.

